# Impact of Participatory Health Research: A Test of the Community-Based Participatory Research Conceptual Model

**DOI:** 10.1155/2018/7281405

**Published:** 2018-04-24

**Authors:** John G. Oetzel, Nina Wallerstein, Bonnie Duran, Shannon Sanchez-Youngman, Tung Nguyen, Kent Woo, Jun Wang, Amy Schulz, Joseph Keawe‘aimoku Kaholokula, Barbara Israel, Margarita Alegria

**Affiliations:** ^1^University of Waikato, Hamilton 3240, New Zealand; ^2^University of New Mexico, Albuquerque, NM 87131, USA; ^3^University of Washington, Seattle, WA 98195, USA; ^4^University of California San Francisco, San Francisco, CA 94115, USA; ^5^NICOS Chinese Health Coalition, 1208 Mason St., San Francisco, CA 94108, USA; ^6^California Institute of Integral Studies, 1453 Mission St., San Francisco, CA 94103, USA; ^7^University of Michigan, Ann Arbor, MI 48109, USA; ^8^University of Hawaii, Honolulu, HI 96822, USA; ^9^Massachusetts General Hospital and Harvard University, Boston, MA 02114, USA

## Abstract

**Objectives:**

A key challenge in evaluating the impact of community-based participatory research (CBPR) is identifying what mechanisms and pathways are critical for health equity outcomes. Our purpose is to provide an empirical test of the CBPR conceptual model to address this challenge.

**Methods:**

A three-stage quantitative survey was completed: (1) 294 US CBPR projects with US federal funding were identified; (2) 200 principal investigators completed a questionnaire about project-level details; and (3) 450 community or academic partners and principal investigators completed a questionnaire about perceived contextual, process, and outcome variables. Seven in-depth qualitative case studies were conducted to explore elements of the model not captured in the survey; one is presented due to space limitations.

**Results:**

We demonstrated support for multiple mechanisms illustrated by the conceptual model using a latent structural equation model. Significant pathways were identified, showing the positive association of context with partnership structures and dynamics. Partnership structures and dynamics showed similar associations with partnership synergy and community involvement in research; both of these had positive associations with intermediate community changes and distal health outcomes. The case study complemented and extended understandings of the mechanisms of how partnerships can improve community conditions.

**Conclusions:**

The CBPR conceptual model is well suited to explain key relational and structural pathways for impact on health equity outcomes.

## 1. Introduction

Community-based participatory research (CBPR) and other forms of community engaged (CEnR) and participatory health research (PHR) are viewed as critical approaches for improving health and health inequity in ethnic/racial minority, underserved, and otherwise vulnerable communities [1–3]. While there is a continuum of community engagement, this paper will use “CBPR” to encompass PHR (used more internationally) and CEnR projects that espouse collaborative practices and values. Further, CBPR promotes implementation of innovative, culturally appropriate, and evidence-based interventions that enhance translation of research findings for community and policy change [4, 5]. As a collaborative research approach, CBPR equitably involves community and academic partners, recognizes the unique strengths of each, shares leadership and resources, addresses health problems important to the community, and uses information gained for community benefit [1, 2]. Supported by communities, CBPR seeks to collaboratively develop research knowledge, mutual trust, culturally centered research methods, sustainable interventions, and community capacity and change power relations among academics, policy makers, community members, and other stakeholders [6–8].

While there is evidence of CBPR promoting positive outcomes, the science and understanding of why it works is in its nascency [9–11]. The main challenge in evaluating and theorizing CBPR is identifying what aspects are critical for interventions and health improvement. Jagosh and colleagues [3] noted that this involves understanding whether context (e.g., cultural nuances), partnership (e.g., degree of cooperation), and research interventions, separately or together, are responsible for contributing to intermediate and distal health outcomes. Given the claim that CBPR brings together individuals and organizations to address unequal distribution of social determinants that contribute to health inequities [12–14], examining how these dynamics work together is critical to understand the added value of CBPR in achieving social justice.

**Figure 1:**
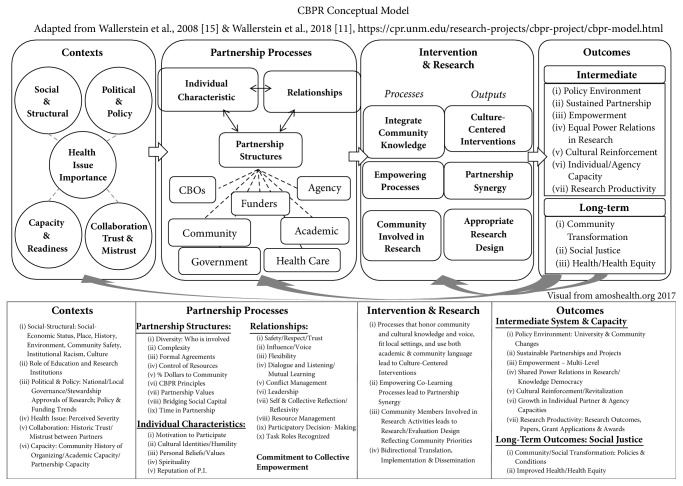
CBPR conceptual model.

Three recent sets of studies advance the science of CBPR. First, Wallerstein and colleagues [15] introduced a CBPR conceptual model with four domains: context, partnership dynamics, research/intervention, and outcomes. The model drew upon prior research [16], extensive literature reviews, a survey of CBPR practitioners, and consultation with a national advisory board of academic and community CBPR experts [15]. It represents visually a flow of domains and attributes that play a role in research and health outcomes. Context includes socioeconomic-cultural characteristics, governance and policy trends, historical collaborations, university and community capacities, and the health issue being researched. Contextual factors provide a backdrop for partnership dynamics, that is, on partnership structures and members and on relationships, including how they are managed and strengthened. If partnering practices are effective, then they shape both intervention and research design, which reflect mutual learning and partner synergy or ability to work together effectively. Finally, the model indicates that CPBR intervention/research processes produce intermediate outcomes such as systems or capacity changes and distal outcomes such as improved community health equity. The original model has undergone community consultations to assess face validity [17], and iterative updates based on our team's research are also used in this article [18] in addition to international translations and applications.  Figure 1 provides the latest iteration of the model.

Second, Khodyakov and colleagues [19, 20] explained how partnership characteristics result in several outcomes among projects focused on mental health and substance abuse issues. The authors surveyed 62 community and academic leaders from 21 federally funded research centers focusing on mental health and completed full-length interviews for 23 projects. They found that community engagement in research and partnership size affect partnership functioning; partnership functioning influences partnership synergy; and partnership synergy positively affects outcomes such as capacity building and community outcomes.

Third, Jagosh and colleagues [3, 10] examined how pathways of trust and commitment to power-sharing in CBPR support sustained collaboration towards health improvement and community transformations. Based on a realist review of literature and interviews with 24 CBPR investigators, they argued that partnership synergy is developed through trust, which has ripple impacts on culturally appropriate research, project sustainability, capacity development, system-changes, and population health outcomes.

While all three sets of studies contribute to the science of CBPR, the CBPR conceptual model is more comprehensive in its coverage by including multiple domains of context, intervention/research, partnership practices, and outcomes [15]. Conceptually, this model embeds health outcomes in local conditions and histories and in broader sociopolitical systems, which shape relationships between partners, and place CBPR/PHR strategies within social justice goals [13, 17, 21]. It further provides a concrete framework for understanding CBPR contexts and dynamics and their impact on research processes and outcomes. This model, therefore, is well-suited for addressing a key gap in CPBR/PHR literature; that is, to theoretically and empirically explain how contexts, partnership practices, and research/intervention engagement factors contribute to broad-based CBPR and health outcomes.

**Figure 2:**
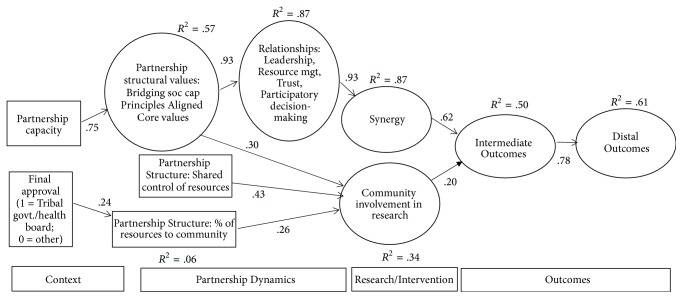
Empirical test of CBPR conceptual model.* Note*. Only significant paths (*p* < or = .01) are displayed.

The purpose of this study is to provide an empirical test of the CBPR conceptual model to better understand the mechanisms for impact on research results, community conditions, and health equity. We could not include every variable from Figure 1 and we derived a model for testing (see Figure 2). We hypothesized that the exogenous contextual variables would shape the partnership structures and dynamics. Further, we hypothesized that the partnership dynamics would be associated with synergy, which in turn is associated with intermediate and then distal outcomes. We also hypothesized that partnership structures and dynamics would be associated with community involvement in research, which in turn is associated with intermediate and then distal outcomes.

## 2. Methods

To test the model, we used data from our Research for Improved Health (RIH) study of 200 US CBPR projects [22]. As a mixed-method design, the sample was drawn from the National Institutes of Health RePORTER database of federally funded CBPR projects. Selected projects completed a cross-sectional Internet survey, paired concurrently with seven diverse qualitative case studies [18]. In this analysis, we examine the fit of the survey data to the conceptual model using structural equation modelling. We also use one of the case studies to illustrate mechanisms of CBPR that complement and extend understanding of the model. IRB approval was provided by two universities and supported by the Indian Health Service review board.

### 2.1. Internet Survey

#### 2.1.1. Research Design and Sampling

The research design included three stages of a cross-sectional survey of federally funded CBPR partnered projects in 2009. Methods are described briefly here, and in depth elsewhere [18, 23, 24]. Phase one involved selecting 294 CPBR projects in 2009 from US databases through an extensive search strategy. Secondly, we sent out a key informant Internet survey (KIS) to principal investigators or project directors (PI) in 2011, with 200 (68.0%) respondents, who also identified up to four partners (three community and one academic) to participate in the community engagement survey (CES).

Thirdly, the CES was sent to 404 partners and 200 PIs in 2012; 450 in total participated: 312 partners (77.2%) and 138 PIs (69.0%). The CES sample included 272 White, non-Hispanic, 37 American Indian/Alaska Native, 37 African American, 32 Hispanic, 28 Asian/Pacific Islander, and 23 mixed race or other; 73 male and 205 female; and 194 community partners and 118 academic partners.

#### 2.1.2. Measures

Table 1 presents descriptive information of measures used in this study including relationship to each domain and construct in the model.  Table 2 presents the original items, scaling, and Cronbach alphas of the measures. Prior studies provide evidence of validity and psychometric properties of the measures including internal consistency and factorial and construct validity [24, 25].

For context, we included in the CES a measure of* partnership capacity* based on a prior measure [26]. The governance context measure in the KIS was* final approval* created by the research team, who provided approval of participation in this research project on behalf of the community, with six response items recoded to tribal government/health board or other. Two other KIS items were* percentage of resources* provided to the community and* shared control of resources* (in-kind, financial, personnel) [23].

Partnership dynamics, measured by the CES, included three broad categories: partnership structures, relationships, and community engagement in research.* Partnership structures* included a prior measure of partner values [27] and two measures created by the research team: principles of CPBR (partner focus) and bridging social capital [24].* Relationships* included leadership, resource management, participatory decision-making [20], trust [28], and (dis)respect, participation, and cooperation [29].* Community engagement in research* (CER) was measured from a prior scale [20] with three subscales: background research, data collection, and analysis and dissemination.

Perceived outcomes, measured in the CES, included the* proximal outcome* of partnership synergy from a previous scale [20];* intermediate outcomes* of three prior scales—personal and agency capacity building [20] and sustainability [24]; and* distal outcomes* of a community transformation scale [20] and a single item measuring improvement in community health [24].

#### 2.1.3. Data Analysis

Data analysis was based on project-level data. Specifically, the CES responses were averaged across the project to create a single score because there was a high level of agreement among the partners within any given partnership about the outcomes ranging from .75 to .88 on a measure of consensus of responses [30]. Analysis of the latent structural model was completed using SPSS AMOS 23.0. The analysis was completed with means and intercepts estimated for missing values using maximum likelihood. There was a small amount of missing data determined to be missing at random. The model was assessed using four fit indices: *χ*^2^ to df ratio (*χ*^2^/df), comparative fit index (CFI), Tucker-Lewis index (TLI), and root mean square error of approximation (RMSEA): CFI and TLI ≥ .90, RMSEA ≤ .08, and *χ*^2^/df ≤ 2.0.

### 2.2. Case Study Methods and Analysis Design

The RIH qualitative arm sought complementary and distinct knowledge on CBPR pathways in the model, specifically asking how contexts* interact *with partner perceptions and how partnership practices over time contribute to the range of outcomes in the model. Concurrent with the survey, we implemented an iterative parallel methodology [31], especially during analysis, using the transformational lens of advancing equity [32]. We chose a purposefully diverse sample of seven case studies, by being urban/rural, geography, health issue, and racial/ethnic or other social identities, for example, the deaf community. Methods (fully described elsewhere [18]) included document review; on-site visits, with individual academic and community interviews, focus groups, meeting observations, and partnership historical timelines; and a brief survey (instruments at [https://cpr.unm.edu/research-projects/cbpr-project/research-for-improved-health.html]). Using ATLAS.ti, we coded transcripts following the model constructs and first triangulated themes with the SEM scales, confirming the importance of context partnership capacity, resource sharing, relational dynamics, CER, synergy, and agency capacity and health outcomes. Secondly, we coded on themes not included in the survey, such as sociocultural historical contexts, trajectories of time and impact, and motivation and actions of partners towards outcomes, which allowed us to add developmental theorizing.

Due to space constraints, this paper reports on one illustrative case study to illuminate pathways and mechanisms in the model. This project was National Institute of Cancer- (NCI-) funded research to test the effectiveness of lay health workers (LHW) to increase colorectal cancer screening among Chinatown immigrants, given inequity in this cancer [33, 34]. The primary partnership was between the University of California San Francisco (UCSF), San Francisco State University, and NICOS Chinese Health Coalition, a community organization; partners also included the Chinatown Health office and AANCART, an NCI-network to address Bay Area Asian-American cancer inequities. Specific data collection included a 2.5-day visit, 11 stakeholder interviews, partnership focus group, historical timeline, and brief partner surveys. Transcripts were transcribed, coded, and consolidated into narratives, which were returned to the partnership for verification, editing, and cointerpretation.

## 3. Results

### 3.1. Latent Structural Model

Prior to testing the latent structural model, the measurement model was examined. The overall measurement model provided a good fit to the data; *χ*^2^  (236, *N* = 161) = 438.97, *p* < .001, CFI = .93, TLI = .91, and RMSEA = .07. To achieve this fit, we had to remove three scales: change in power relations, principles (community focus), and influence as these scales had significant overlap with other scales in the model. We chose to remove these scales rather than try to include items in the retained scales as we had established distinctness of the scales in prior testing.

Figure 2 illustrates the model and the significant paths among latent variables to include the effect sizes (e.g., variance accounted for). The model achieved reasonable fit. *χ*^2^ (315, *N* = 161) = 542.95, *p* < .001, CFI = .92, TLI = .90, and RMSEA = .07. Table 1 includes the description of, and relationships among, the constructs in the model. The contextual variables were associated with partnership structures and dynamics although in unique ways. Partnership capacity was positively associated with partnership structural values. Partnership structural values were then positively associated with relationships and CER. Governance, as final approval given by a health board/tribal government, was associated with a greater percentage of resources to the community. Greater percentage of resources and shared control of resources were associated with CER.

Relationships were strongly and positively associated with synergy. Both CER and synergy were positively associated with intermediate outcomes, which were strongly and positively associated with distal outcomes.

### 3.2. Case Study

The qualitative data below offers support for quantitative findings, as well as new findings of the relationships between CBPR model domains. Given space limitations, single exemplary quotes are provided even though multiple community and academic partners supported each theme.

Qualitative context data provided distinct information about community inequities and consequent effect on research participation.

40% of Chinese households are linguistically isolated… no one over the age of 14 speaks English well or at all. [As] new immigrants, they have a lower standard of living…If they are people who recently came to the country, especially from mainland China, they're really sceptical about research…They might think that the government is trying to get something from them.

Yet understanding cultural foundations as assets was also important.

To understand why we run things certain ways, a person would need to understand Chinese culture... Nutrition is of great interest…they go to herbalists to make soup to get better.

These elements exemplify the need to focus on the broader context of the community that the partnership needs to understand and operate within. These broader contexts of community, including how the role of nutritional health could add to research messaging, were not captured in the surveys.

The understanding of a survey context variable, partnership capacity, was strengthened with information about the existing capacity of NICOS, as a highly regarded community organization and key community partner, to provide proxy trust for the academics. With a subcontract from UCSF, as an important structural construct in the model of sharing resources, NICOS became the de facto implementer and bridge, hiring LHWs and a research coordinator, who worked closely with the UCSF coordinator. Having an influential community partner facilitated successful implementation of the grant, especially through collaborative structures (i.e., the second domain of the model).

The roles of subcommittees of both academic and community members, not included in the surveys, were noted as important bridging mechanisms to good relationships and effective collaborative work.

One of the things that makes us have such a good working relationship is the sub-teams; the translation sub-team, because we're all Chinese speakers. We have a lot of fun, because sometimes things can be translated in a really funny way…..And we have a lot of laughs…just trying to figure out what's the right way…After meetings we go eat lunch together. That really helps in developing a good working relationship.

Since many academic team members were multilingual/multicultural, they used this culturally centered bridging capacity to cocreate the intervention and research materials, seen as cultural fit in the third domain of the model.

… all the materials, I would say it's scrutinized by a group of Chinese people. Like we spend so much time on translation and just reviewing whether the pictures are culturally appropriate, the wordings, everything…It's unbelievable how much time we spend”…. “Like they would say a common belief among the Chinese about colon cancer; and then instead of saying ‘don't do that; that doesn't work,'….in our flip chart, they don't approach it that way. They say, ‘It's good you are doing something to promote your health.' The best way…is to combine the Chinese and the western ….This is something I've never seen before.

Respect for different expertise of the partners was also apparent in how partners talked about CBPR and on their working together well, defined in the model and literature as synergy.

CBPR really opens up the communication channels … not everyone is a trained researcher, but we all have the same goal…..and that really influences how we work together. We all have different expertise…We look to UCSF for all their research-related questions we have; and NICOS is more the community expert…And San Francisco State, their specialty is… traditional Chinese medicine…For different issues, we go to different people. And most of the time we respect the other party's expertise, and we accept what they suggest.

The use of structural features of subcommittees and shared resources helped in creating synergy and cultural centeredness and enable intermediate and distal outcomes.

Outcomes of the trial confirmed intervention effectiveness on knowledge and screening [35]. In addition to research outcomes, the strength of synergy meant that social outcomes were also embraced by the academics, facilitated by a new favourable political environment.

I think the community is also highly activated... You know the mayor now is Chinese…There's a very powerful awakening in the Chinese community politically.

This meant for NICOS that not only did they gain agency capacity in research, but there was a window of opportunity for their broader LHW workforce development agenda. This illustrates how intervention outcomes can foster political opportunities for partnerships to promote health equity.

It won't affect the project directly, except if we decide to take this workforce issue as far as we want, there might be more sympathetic ears at certain places. We just found obviously that when you go into a Chinese-American leader and say, “Well, this a need for the community,” they tend to grasp it a little bit quicker than having to explain to someone who's not from the community.

In sum, qualitative data deepened an understanding of temporal pathways of how community and partnership capacity and the structures of subcommittees and shared resources interacted with CBPR-driven mutual relationships and culture-centered interventions to strengthen synergy and promote health and social equity outcomes, including potential feedback loops to change contexts.

## 4. Discussion

The purpose of this study was to provide a mixed-method assessment of the domains and pathways linking components of the CBPR conceptual model to demonstrate the transformational impact of participatory health research on health and social equity. The model was validated by a robust mixed-method data set from a large US sample of CBPR projects across different communities and health conditions. It is the first attempt at using a latent variable structural model to examine components of CBPR.

There are two key parts to the contextual and relational dynamics domains and their respective impacts demonstrated by the structural equation model. One is the shared governance structure of the projects in terms of approvals, resource sharing, and resource control (i.e., structural pathway). These factors ensure community engagement and representation in the research, facilitating community stewardship, and making sure that the community benefits [25]. The other factor is the partnership having capacity and high quality partnership practices, having resources and skills to interact with principles of mutual learning and respect (i.e., relational pathway) [16, 36, 37]. These elements collectively shape partnership synergy given the significant paths in the model, as synergy is associated with effective relational dynamics [10, 20, 38, 39]. Further, synergy and CER mediate context and partnership dynamics with the intermediate and distal outcomes, consistent with other research [10, 20].

The qualitative results reinforce this model and provide distinct findings of how relationships are strengthened and how the model moves across time towards outcomes, beyond specific grant aims. Qualitative findings demonstrate depth and specificity in the conceptual relationships and extend the model particularly in terms of context and feedback loops. For example, case study data provide a deeper understanding of context, especially identifying the sociohistorical risks and assets in which the partnership is situated. Community contexts then shape the development of trust and navigating structural and relational dynamics [18]. These findings also provide more depth to understanding how partnership structures and relationships interact in order to create synergy. In this particular case, the respected community organization was widely credited as strengthening synergy by bridging academic and community members. Finally, the case study was able to illustrate how various outcomes of the project provide feedback to context and relational dynamics. This dynamism is reflective of CBPR and helps to overcome limitations of the cross-sectional survey.

A major implication of this study is the impact that CBPR context and dynamics has on intermediate and distal outcomes. This study demonstrates with mixed-method data that the nature of partnership dynamics within a particular context has effects on a variety of capacity building, community transformation, and community health outcomes through partnership synergy as demonstrated by the positive and significant paths in structural equation model from context and partnering processes to outcomes. A key goal of many using CBPR, and other forms of PHR, is to contribute to social justice and public health and this study provides strong evidence that partnering processes matter for health and social equity outcomes [13, 14, 21].

The study also has some limitations. The survey is cross-sectional, with perceptual measures of dynamics and outcomes. The case study helps address this limitation as it is from a different sample and is avoiding same sample bias. While also based on people's perceptions, community and academic stakeholders were deeply engaged with strong awareness of their practices and impact of their work on the community. However, future research should also examine longitudinal and actual outcomes resulting from CBPR processes to assess the extent to which perceived and observed indicators work in similar or distinct ways, and whether one may be more predictive of outcomes than the other, and, if so, under what conditions.

## 5. Conclusion

This study sought to provide an empirical test of the CBPR conceptual model to advance the science of CBPR and other forms of PHR. The mixed-methods findings from a robust data set provide some empirical support for specific domains of the model, with pathways identified through both quantitative modelling and qualitative data. Qualitative data additionally offered insights into how context and partnering practices influence each other across time towards partnership effectiveness. The model can be used as a theoretical and evaluation tool to help enhance the practice of the many forms of participatory health research and holds much promise for achieving health equity and improving the health of communities. These approaches provide the opportunities for communities to codevelop and thus allow for holistic self-determined interventions that reflect the life experiences, values, and goals of the community. The current study helps to illustrate key contextual and partnering processes to enable this type of impact.

## Figures and Tables

**Table 1:** Descriptive information for constructs.

Latent variable	Definition	Included items or scales	M	SD	1	2	3	4	5	6	7	8	9
(1) Partnership capacity	Project has resources to achieve its aim	Partnership capacity	4.34	.38	1.0								

(2) Final approval	Who approved the project on behalf of the community	Final approval (tribal govt./health board or other)	.24	.43	.02	1.0							

(3) Control of resources	Whether resources were controlled by community, academic, or both	In-kind, financial, and hiring	2.02	1.00	.11	−.12	1.0						

(4) % of resources to community	The percentage of overall financial resources that went to the community	Percent of resources to community	36.08	25.67	−.08	.24^*∗*^	.03	1.0					

(5) Partnership structural values	Principles, values, and people for organizing the partnership	Partnership values, alignment with CBPR principles—partner focus, bridging social capital	13.05	1.24	.73^*∗*^	−.07	.10	−.16	1.0				

(6) Relationships	Nature and quality of the interaction among the partners	Leadership, resource management, participatory decision-making, trust, respect, participation, cooperation	24.11	2.60	.59^*∗*^	−.09	.15	−.12	.78^*∗*^	1.0			

(7) Community involvement in research	Extent to which community members are involved in research	Background research, data collection, data analysis & dissemination	6.57	1.24	.14	−.07	.41^*∗*^	.22^*∗*^	.21^*∗*^	.22^*∗*^	1.0		

(8) Synergy	Ability to work effectively	Partnership synergy	4.45	.49	.61^*∗*^	.04	.15	−.01	.75^*∗*^	.82^*∗*^	.29^*∗*^	1.0	

(9) Intermediate	Intermediate outcomes	Agency capacity building, personal capacity building, and sustainability	10.83	1.74	.52^*∗*^	−.05	.24^*∗*^	.05	.61^*∗*^	.56^*∗*^	.34^*∗*^	.59^*∗*^	1.0

(10) Distal	Long-range outcomes	Community transformation, community health improvement	6.41	1.41	.42^*∗*^	.01	.24^*∗*^	.09	.47^*∗*^	.38^*∗*^	.20^*∗*^	.43^*∗*^	.59^*∗*^

^*∗*^ *p* < .01.

**Table 2:** Scale items and Cronbach's alphas for measures.

Category	Scale	Items	Response scale	Cronbach's alpha
Context	Partnership capacity	(1) Skills and expertise (2) Diverse membership (3) Legitimacy and credibility (4) Ability to bring people together for meetings and activities (5) Connections to political decision-makers, government agencies, and other organizations/groups (6) Connections to relevant stakeholders	1 (not at all) to 5 (to a great extent)	0.78
	Final approval	(1) Who made the final decision to approve participation in this research projects on behalf of the community? Choose all that apply	(1) Agency leader, representative, board, or staff, (2) tribal/local government or health board/public health office, (3) individual community member(s), (4) project advisory board, (5) no community decision; individual research participants give consent	N/A

Partnership structures	Shared control of resources	(1) Which partner (academic, community, or both) hires personnel on the project? By community partners we mean agencies, organizations, tribal communities, health departments, individuals, or other entities representing communities By academic partners we mean university or research institutions (2) Who decides how the financial resources are shared? (3) Who decides how the in-kind resources are shared?	(1) Community; (2) academic; (3) both	N/A
	Percentage of resources to the community	(1) Thinking of the overall budget, how are the project's financial resources divided among community partners and academic partners?	% to community	N/A

Partnership structural values	Bridging social capital	(1) Does the *community* research team have the knowledge, skills, and confidence to interact effectively with the academic researcher team? (2) Does the *academic* research team have members who are from a similar cultural background as the community research team? (3) Overall, does the *academic* research team have the knowledge, skills, and confidence to interact effectively with the community research team?	1 (not at all) to 5 (to a great extent)	0.69
	Alignment with CBPR principles: partner focus	(1) This project builds on resources and strengths in the community (2) This project emphasizes what is important to the community (environmental and social factors) that affect well-being (3) This project views community-engaged research as a long term process and a long term commitment (4) This project fits local/cultural beliefs, norms, and practices	1 (not at all) to 5 (to a great extent)	0.82
	Core values	(1) Members of our partnership have a clear and shared understanding of the problems we are trying to address (2) There is a general agreement with respect to the mission of the partnership (3) There is general agreement with respect to the priorities of the partnership (4) Members agree on the strategies the partnership should use in pursuing its priorities	1 (strongly disagree) to 5 (strongly agree)	0.89

Relationships	Participation	(1) We showed positive attitudes towards one another (2) Everyone in our partnership participated in our meetings (3) We listened to each other	1 (strongly disagree) to 5 (strongly agree)	0.78
	Cooperation	(1) Arguments that occurred during our meetings were constructive (2) When disagreements occurred, we worked together to resolve them (3) Even though we did not have total agreement, we did reach a kind of consensus that we all accept	1 (strongly disagree) to 5 (strongly agree)	0.83
	Respect	(1) There were disrespectful remarks made during the conversation (2) There was hidden or open conflict and hostility among the members (3) The way the other members said some of their remarks was inappropriate	1 (strongly disagree) to 5 (strongly agree)	0.83
	Trust	(1) I trust the decisions others to make about issues that are important to our projects (2) I am comfortable asking other people to take responsibility for project tasks even when I am not present to oversee what they do (3) I can rely on the people that I work with on this project (4) People in this group/community have confidence in one another	1 (strongly disagree) to 5 (strongly agree)	0.86
	Participatory decision-making	(1) Feel comfortable with the way decisions are made in the project (2) Support the decisions made by the project team members (3) Feel that your opinion is taken into consideration by other project team members (4) Feel that you have been left out of the decision making process	1 (never) to 5 (always)	0.83
	Leadership	(1) Taking responsibility for moving the project forward (2) Encouraging active participation of academic and community partners in the decision-making (3) Communicating the goals of the project (4) Working to develop a common language (5) Fostering respect between partners (6) Creating an environment where differences of opinion can be voiced (7) Resolving conflict among partners (8) Helping the partners be creative and look at things differently (9) Recruiting diverse people and organizations into the project (10) Providing orientation to new partners as they join the project	1 (very ineffective) to 5 (very effective)	0.94
	Resource management	(1) The team's financial resources (2) The team's in-kind resources (3) The team's time	1 (makes poor use) to 5 (makes excellent use)	0.86

Research/intervention	Community involvement in research: background research	(1) Developing community-based theories of the problem or intervention (2) Grant proposal writing (3) Background research (4) Choosing research methods (5) Developing sampling procedures	1 (community partners did not participate in this activity) to 3 (community partners were actively engaged in this activity)	0.81
	Community involvement in research: data collection	(1) Recruiting study participants (2) Implementing the intervention (3) Designing interview and/or survey questions (4) Collecting primary data	1 (community partners did not participate in this activity) to 3 (community partners were actively engaged in this activity)	0.69
	Community involvement in research: analysis & dissemination	(1) Interpreting study findings (2) Writing reports and journal articles (3) Giving presentations at meetings and conferences	1 (community partners did not participate in this activity) to 3 (community partners were actively engaged in this activity)	0.82
	Partnership synergy	(1) Develop goals that are widely understood and supported in this partnership (2) Develop strategies that are most likely to work for your community or stakeholders as a whole (3) Recognize challenges and come up with good solutions (4) Respond to the needs and problems of your stakeholders or community as a whole (5) Work together as a team	1 (not at all) to 5 (to a great extent)	0.90

Intermediate outcomes	Personal capacity building	(1) Enhanced my own reputation (2) Increased utilization of my expertise or services (3) Increased my ability to acquire additional financial support	1 (not at all) to 5 (to a great extent)	0.80
	Agency capacity building	(1) Enhanced the agencies' reputation (2) Enhanced the agencies' ability to affect public policy (3) Increased utilization of agencies' expertise or services	1 (not at all) to 5 (to a great extent)	0.87
	Sustainability of partnership/project	(1) I am committed to sustaining the community-academic relationship with no or low funding (2) This project is likely to continue forward after this funding is over (3) Our partnership carefully evaluates funding opportunities to make sure they meet both community and academic partners' needs	1 (strongly disagree) to 5 (strongly agree)	0.71

Distal outcomes	Community transformation	(1) Resulted in policy changes (2) Improved the overall health status of individuals in the community (3) Resulted in acquisition of additional financial support (4) Improved the overall environment in the community	1 (strongly disagree) to 5 (strongly agree)	0.79
	Health outcomes: community health improvement	(1) Overall, how much did or will your research project (insert name) improve the health of the community?	1 (not at all) to 5 (a lot)	N/A
